# Supplementary material for: SPHIRE-crYOLO is a fast and accurate fully automated particle picker for cryo-EM
Source: Commun Biol. 2019 Jun 19;2:218. doi: 10.1038/s42003-019-0437-z (PMC6584505; doi:10.1038/s42003-019-0437-z)
Supplement: Supplementary file 1 — Description of Supplementary Data [file 42003_2019_437_MOESM1_ESM.pdf]

Description of Additional Supplementary Files

File Name: Supplementary Data 1
Description: Precision / recall values in crYOLO evaluated on KLH in dependence of the number of training images. First column lists recall values, second column precision values.

File Name: Supplementary Data 2
Description: AUC, recall and precision values for the general training dataset (without simulated data). The values were evaluated with the crYOLO network (column 1-3) and Inception-ResNet (column 4-6).

File Name: Supplementary Data 3
Description: Precision / recall values in crYOLO evaluated on TcdA1 picking with a directly trained model or the general model. First column lists recall values, second column precision values.
