# Supplementary material for: SPHIRE-crYOLO is a fast and accurate fully automated particle picker for cryo-EM
Source: Commun Biol. 2019 Jun 19;2:218. doi: 10.1038/s42003-019-0437-z (PMC6584505; doi:10.1038/s42003-019-0437-z)
Supplement: Supplementary file 5 — Supplementary Figure [file 42003_2019_437_MOESM5_ESM.docx]

**
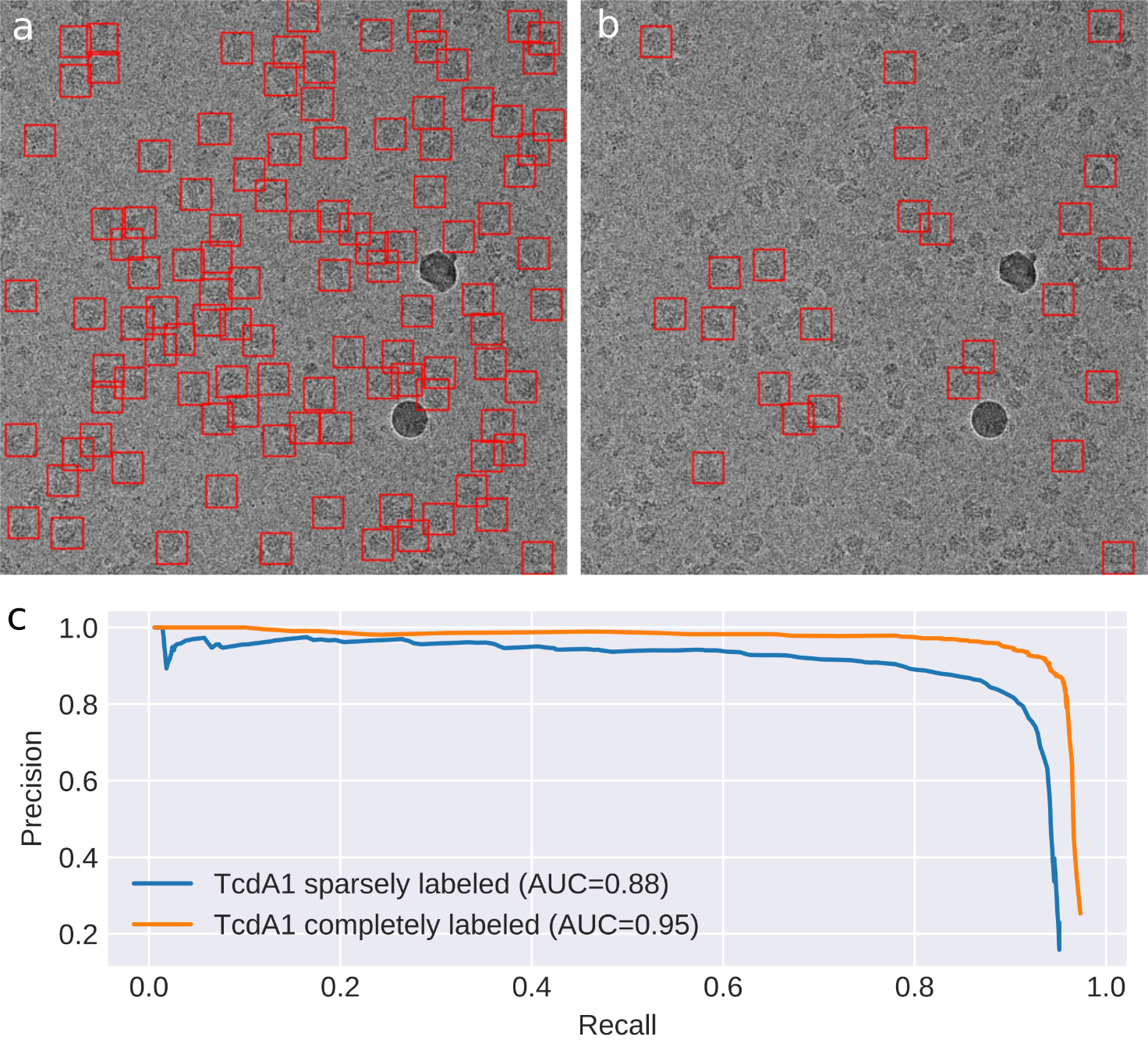
**

**Supplementary Figure 1: Evaluation of crYOLO on sparse training data.** Comparison of **(a)** completely labeled training data with **(b)** sparsely labeled training data. The training set comprises 10 micrographs of TcdA1 with 1100 particles in the case of completely labeled micrographs, and 195 particles in the case of sparsely labeled micrographs. **c)** Precision-recall curves for TcdA1 picked with either a network trained on 10 micrographs with particles picked to completion (orange) or picked sparsely (blue).
